# Addition of anaerobic electron acceptors to solid media did not enhance growth of 125 spacecraft bacteria under simulated low-pressure Martian conditions

**DOI:** 10.1038/s41598-020-75222-2

**Published:** 2020-10-26

**Authors:** Petra Schwendner, Mary-Elizabeth Jobson, Andrew C. Schuerger

**Affiliations:** Space Life Sciences Lab, University of Florida, 505 Odyssey Way, Exploration Park, Merritt Island, FL 32953 USA

**Keywords:** Microbiology, Planetary science

## Abstract

To protect Mars from microbial contamination, research on growth of microorganisms found in spacecraft assembly clean rooms under simulated Martian conditions is required. This study investigated the effects of low atmospheric pressure on the growth of chemoorganotrophic spacecraft bacteria and whether the addition of Mars relevant anaerobic electron acceptors might enhance growth. The 125 bacteria screened here were recovered from actual Mars spacecraft. Growth at 7 hPa, 0 °C, and a CO_2_-enriched anoxic atmosphere (called low-PTA conditions) was tested on five TSA-based media supplemented with anaerobic electron acceptors. None of the 125 spacecraft bacteria showed active growth under the tested low-PTA conditions and amended media. In contrast, a decrease in viability was observed in most cases. Growth curves of two hypopiezotolerant strains, *Serratia liquefaciens* and *Trichococcus pasteurii*, were performed to quantify the effects of the added anaerobic electron acceptors. Slight variations in growth rates were determined for both bacteria. However, the final cell densities were similar for all media tested, indicating no general preference for any specific anaerobic electron acceptor. By demonstrating that a broad diversity of chemoorganotrophic and culturable spacecraft bacteria do not grow under the tested conditions, we conclude that there may be low risk of growth of chemoorganotrophic bacteria typically recovered from Mars spacecraft during planetary protection bioburden screenings.

## Introduction

Protecting solar system bodies from contamination by Earth life not only allows the preservation of extraterrestrial habitats in their natural state but also is a precaution to avoid contamination in places where life might exist. For Mars, Special Regions have been defined as environments “within which terrestrial organisms are likely to propagate” or “any region which is interpreted to have a high potential for the existence of extant Martian life”^1^. Life-detection experiments on future Mars landers may target Special Regions because local conditions are likely more conducive to life than other terrains^2,3^. In order to protect potential Special Regions—that might be identified in the future—from spacecraft contamination, and to potentially search for an extant Mars microbiota, research to characterize microbial survival, metabolism, growth, and evolution must be conducted under relevant Martian conditions.

Prior to a launch, microbial surveys are completed for spacecraft and in spacecraft assembly facilities (SAFs) in which the landers and rovers are assembled^4–6^. Space fairing nations have established standard protocols for the enumeration of biological burden on Mars-bound spacecraft (see ECSS-Q-ST-70-58C)^7,8^. For current missions to Mars, the bioburden constraints are derived from quantitative studies of the Viking spacecraft in the mid-1970s. Since then, these guidelines have been routinely implemented, clean rooms and their payloads meticulously cleaned and screened, and microbial species found on spacecraft archived^9^. Despite the cleaning efforts, the current guidelines allow a certain amount of bacteria to be present on a Mars-bound spacecraft where it mostly encounters harsh and hostile conditions^1,7^.

In order for bacteria on spacecraft to survive and grow on Mars, numerous conditions must be present to permit metabolism, cell proliferation, and adaptation of Earth microorganisms. Various studies have discussed up to 22 biocidal or inhibitory factors that are likely present on Mars (reviewed in^1,10–13^). Recent experiments with bacteria exposed to low-pressure (7 hPa), low-temperature (0 °C), and a CO_2_-enriched anoxic atmosphere (henceforth called low-PTA conditions) have demonstrated that at least 30 bacterial species from 10 genera are capable of metabolism and growth under Mars-relevant low-PTA conditions. The most common bacterial genera capable of growth at low pressures include members from *Bacillus*, *Carnobacterium*, *Clostridium*, *Cryobacterium*, *Exiguobacterium*, *Paenibacillus*, *Rhodococcus*, *Serratia*, *Streptomyces*, and *Trichococcus*^12,14,15^. These hypopiezotolerant microorganisms (def., able to grow at ≤ 10 hPa^16^) inhabit diverse ecological niches including arctic and alpine soils, Siberian permafrost, environmental surface waters, plant surfaces, and seawater; and represent only a minor fraction of the overall microbiota while the vast majority of microorganisms were not able to proliferate even though adequate water and nutrients were provided (e.g.,^15^). However, in these studies, the possibility that microbial species required specific geochemical redox couples or terminal anaerobic electron acceptors under low-PTA conditions for growth was not examined.

The importance for redox couples to provide energy that can drive metabolism under low-PTA conditions is mentioned theoretically as an essential requirement on Mars^12^, but empirical data is lacking. Redox couples provide the energy to power metabolic activity, and in aerobic habitats on Earth, respiration using oxygen as an electron acceptor from the breakdown of organics is an efficient way to produce energy. However, on Mars, the pO_2_ is either low (< 0.13% at the surface) or possibly non-existent (subsurface environments)^1,11^. In order for microorganisms to metabolize and grow in the shallow subsurface on Mars, a different set of redox couples may be required for metabolic activity. In addition, simulated Martian atmospheric pressures of 7 hPa can restrict the capability to metabolize certain organics^17^. To date, bacteria from actual Mars spacecraft and their surrounding SAF clean rooms have not been tested for growth under low-PTA conditions.

The current study investigated two goals. First, with regard to forward contamination, a broad diversity of mesophilic and heterotrophic culturable bacteria isolated from the Viking, Pathfinder, Spirit, Opportunity, Phoenix, Curiosity, and InSight spacecraft were subjected to low-PTA conditions and growth was measured over 28 days. The second objective was to evaluate a range of Mars-relevant geochemical terminal anaerobic electron acceptors to determine if they would enhance microbial activity and growth under simulated low-PTA conditions.

The aims and assumptions for the current study were: (a) test the effect of simulated Martian low pressure of 7 hPa at 0 °C and a CO_2_ atmosphere reflecting a subset of environmental conditions microorganisms might encounter on Mars, (b) that spacecraft microorganisms are shielded from UV irradiation, (c) test for growth of 125 culturable mesophilic bacteria from authentic Mars spacecraft under low-PTA conditions, (d) create a set of ‘conducive’ nutritional and hydrated conditions such that water and nutrient requirements are not limiting in order to focus on the effects of low pressure on bacterial growth, and (e) if none of the bacteria exhibit growth under low-PTA conditions + nutrients + liquid water conditions, then it would be unlikely that they would be able to grow if additional Martian stressors [e.g., salts, low water activity (a_*w*_), extreme diel temperature fluctuations, etc.] were added to the experiments. The results described below are thus a set of experiments in a series of studies exploring the effects of low-pressure environments on the survival, metabolism, growth, and evolution of spacecraft microorganisms under a subset of simulated Martian conditions.

## Material and methods

### Microbial strains

**Figure 1:**
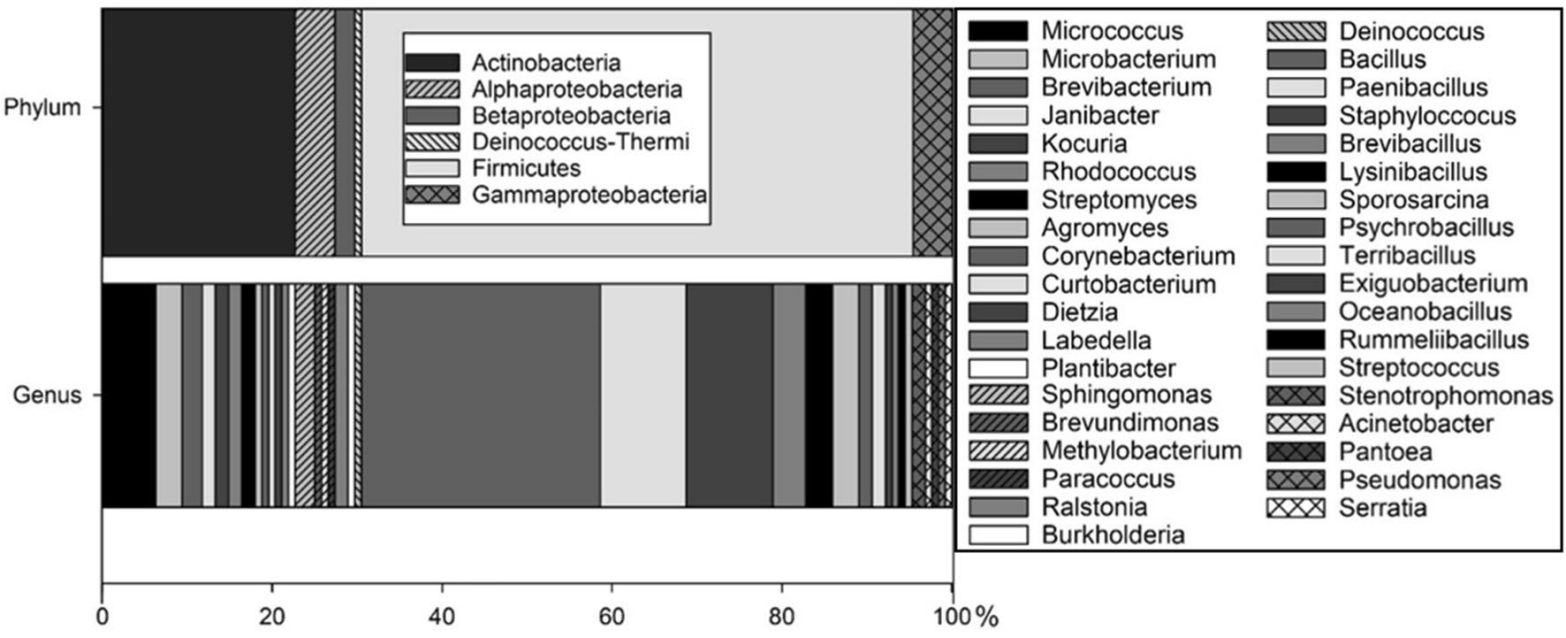
Bacterial diversity of spacecraft-associated microorganisms tested under simulated low-PTA conditions. Phylum and genus levels are displayed as percentage. A full list of bacterial strains tested with supportive metadata can be found in the Table S1.

We surveyed 125 bacteria recovered from six authentic Mars spacecraft including the Viking, Pathfinder, Spirit, Opportunity, Phoenix, InSight and Curiosity platforms (Fig. 1;^9^). The microbial samples were taken during prelaunch activities according to NASA standard protocols^8,18^ and enriched on trypticase soy agar (TSA). The strains were obtained from the Jet Propulsion Laboratory, Pasadena, CA, USA and the Northern Regional Research Laboratory (NRRL) Collection, U.S. Dept. of Agriculture, Peoria, IL, USA. A full list of the spacecraft bacteria can be found in Supplementary Table S1.

**Figure 2:**
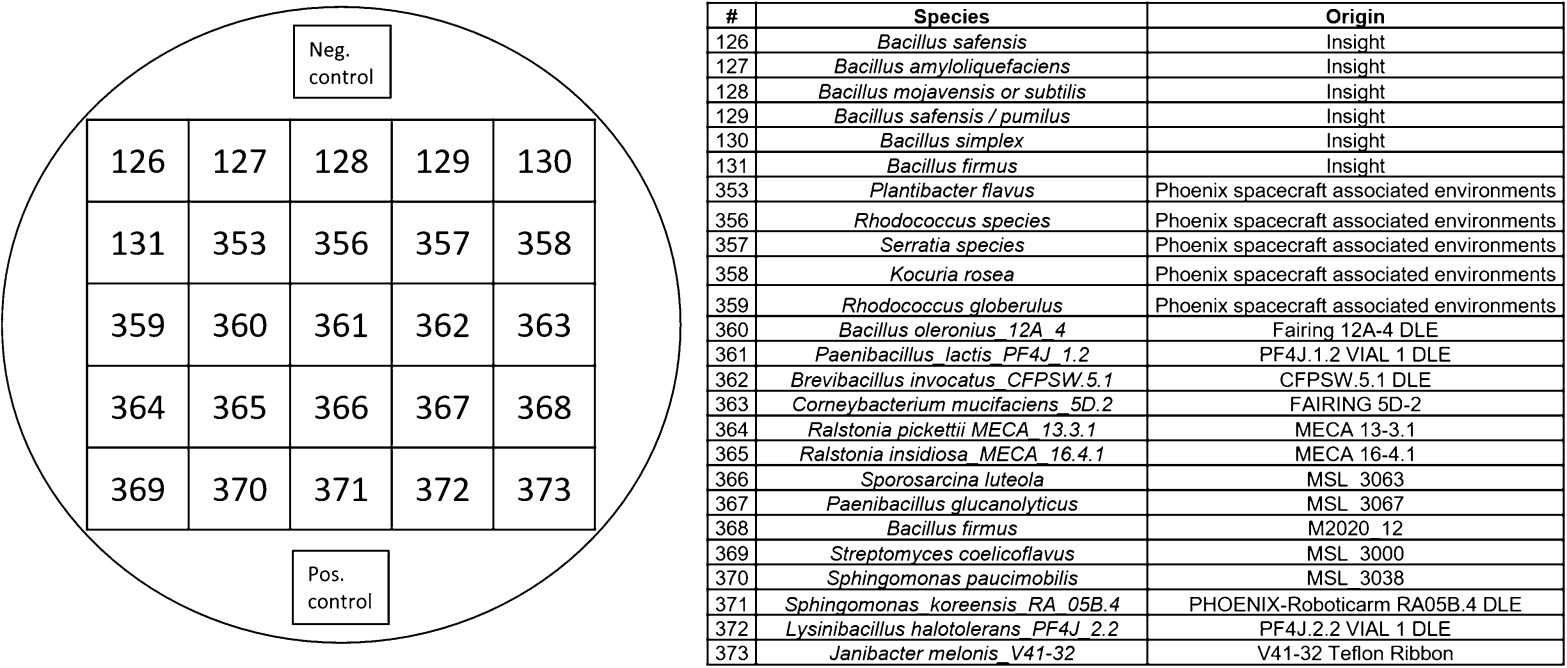
Experimental setup of 25 bacterial strains placed on one TSA plate; plus one negative control (*Bacillus subtilis* 168;^15^) and one positive control (*Serratia liquefaciens* ATCC 27592; from^12^).

The strains were chosen to cover a broad range of culturable chemoorganotrophic bacterial diversity as well as to include genera of known hypopiezotolerant bacteria (from^15^). Representatives from the following 6 phyla were selected: Actinobacteria; Deinococcus-Thermi; Firmicutes; and α-, β-, and γ-Proteobacteria. A total of 37 different genera were tested on six TSA-based media supplemented with diverse anaerobic electron acceptors. An agar-based method was used to simultaneously screen 25 bacterial strains on individual TSA plates including a positive and negative control (Fig. 2; Table S1); respectively, *Serratia liquefaciens* ATCC 27592 as a positive control, and *Bacillus subtilis* 168 as a negative control^12,15^.

### Enrichment conditions

Vegetative cells of all bacteria were prepared on 24-h cultures of TSA incubated at 30 °C. The strains were subjected to the following enrichments conditions and visually checked every 7 days for a total of 28 days: (1) simulated Martian atmospheric pressure conditions at 7 hPa, 0 °C, and a CO_2_-enriched anoxic atmosphere using hypobaric growth chambers (i.e., low-PTA conditions); (2) Control-1: 1013 hPa, 0 °C, and a CO_2_-enriched anoxic atmosphere; (3) Control-2: 1013 hPa, 0 °C, and an Earth-normal atmosphere (pN_2_:pO_2_ at a ratio of 78:21); and (4) Control-3: 1013 hPa, 30 °C, and an Earth-normal atmosphere (pN_2_:pO_2_ at 78:21). The TSA assays were run for only 28 days, and not longer, because the agar-based protocol cannot be easily extended beyond 28 days due to the slow dehydration of the agar at 7 hPa^12,15^. However, the length of time tested here was adequate to identify 30 hypopiezotolerant bacteria from 10 genera in previous work with spacecraft microorganisms^12^ and arctic and permafrost soils^15^.

### Growth under low-PTA conditions

The design and operation of the hypobaric chamber was described previously^12,15^. Briefly, double-thick agar plates (~ 25 mL of TSA) supplemented with the anaerobic electron acceptors were inserted into a 4-L polycarbonate desiccator (model 08-642-7, Fisher Scientific, Pittsburgh, PA, USA) connected to a low-pressure controller (model PU-842, KNF Neuberger, Trenton, NJ, USA). Four anaerobic pouches (model 23-246-378 AnaeroPak System sachets; Mitsubishi Gas Chemical America, Inc. NY, USA;^19^) and an indicator tablet (RT Anaero-Indicator, Mitsubishi Gas Chemical America, Inc.) were added to each desiccator to maintain anoxic conditions. Once the desiccator lid was closed, the low-pressure chamber was flushed for 1–2 min with ultra-high purity CO_2_ gas passed through filter-sterilized (0.22 µm) vent lines. The desiccator was placed in a microbial incubator set at 0 °C and the pressure was reduced stepwise to reach 7 hPa (see^12^ for the depressurization protocol). The low-PTA conditions were maintained for 4 weeks. The desiccator was only vented and opened at 7 day intervals to replace the anaerobic pouches and the indicator tablet, or at additional time points for the determination of growth curves (see below).

### Media assay

The standard medium used was 0.5 × TSA (BD Difco, Becton, Dickinson and Company, Sparks, MD, USA; henceforth just TSA). An organic rich medium was used in order to provide adequate nutrition for the assays that specifically were investigating low-pressure conditions and anaerobic electron acceptors on supporting growth of chemoorganotrophic spacecraft bacteria. All tested spacecraft bacteria were originally recovered on TSA from spacecraft surfaces based on the procedures outlined in the planetary protection protocols^7,8^.

To investigate whether the addition of anaerobic electron acceptors enhanced bacterial growth, the following supplements were added (all weights on a per-liter basis): (1) TSA; (2) TSA + 1 g KNO_3_ (nitrate reduction); (3) TSA + 0.1 g (NH_4_)_2_SO_4_ × 6 H_2_O, 1.5 g Na_2_SO_4_, 1.5 g MgSO_4_ × 6 H_2_O, and 1.5 g sodium lactate (70% v/v; sulfate reduction); (4) TSA + 2.5 g Fe^3+^citrate and 1.5 g sodium lactate (70% v/v) at pH 5.0 (1^st^ iron reduction), (5) TSA + 2.5 g Fe^3+^citrate and 1.5 g sodium lactate (70% v/v) at pH 7.0 (2nd iron reduction); and (6) TSA + 10 ml vitamins (vitamin solution MD-VS, American Type Culture Collection (ATCC), Manassas, VA, USA) + 10 ml mineral solution (trace mineral supplement, ATCC). Bacteria were streaked on all six media in groups of 25 strains plus one positive control (*Serratia liquefaciens* ATCC 27,592;^15^) and one negative control (*Bacillus subtilis* 168;^20^), and incubated at the four different environmental conditions for 28 days. Every 7 days, assay plates were visually inspected for bacterial growth, and then equilibrated back to the diverse test conditions.

### Growth curves

A subset of the 125 spacecraft bacteria screened above was selected for quantitative growth curve assays. Single colonies were taken from 24-h cultures on TSA and mixed in sterile 1 × phosphate buffered saline (PBS). The optical density (OD) of the cell suspensions were measured at 400 nm using a spectrophotometer (Genesys 30 Visible Spectrophotometer, Thermo Scientific, USA), and the suspensions were adjusted as necessary to obtain OD values of 0.007. The resulting cell suspensions (approx. 1 × 10^7^ cells/mL) were pipetted as 2.5-µL drops (i.e., 2.5 × 10^5^ cells/drop) in a five-by-five grid pattern on TSA plates supplemented with the anaerobic electron acceptors listed above. To obtain growth curves of the bacteria on the specific media, approx. 1 × 1 cm squares around the cell-suspension drops were excised from the agar every 3–4 days over the course of 28 days, and processed as follows.

To quantify the bacteria, a sterile scalpel was used to cut out individual droplets removing a thin layer of agar. The extracted agar/cell sections were placed into separate 2 ml sterile microfuge tubes with 1 mL of 1 × PBS buffer. The tubes were vortexed to suspend the bacterial cells and serially diluted in tenfold increments in sterile PBS buffer to determine whether the viable numbers of cells per 2.5 µl drop changed over time. Cell dilutions per sample were processed in 96-well microtiter plates for a series of Most Probable Numbers (MPN) assays^21^ to determine the viable bacterial concentration per milliliter.

### Growth rate analysis

The cell count data were plotted on a logarithmic scale. Based on these graphs, the growth rates of bacteria were determined by the time points representing logarithmic growth. By eliminating extraneous data from the plots, and analyzing only linear functions that best fit the data, growth rates (GR) of each strain/media combination were calculated using the slopes of the linear fitted models for the exponential phases of growth curves. In addition, the doubling times (DT) were determined using the following equation:

$$DT=\frac{\mathrm{ln}2}{r}$$

in which, DT = doubling time, r = the growth rate, given from the slope of the equation for each strain and growth condition tested^22^. Raw data and equations are reported in Table S2.

### a_*w*_, pH, EC, and Eh measurements

All media doped with the various anaerobic electron acceptors were measured for water activity (a_*w*_), hydrogen ion concentration (pH), electrical conductivity (EC), and redox potential (Eh). The a_*w*_, pH, EC, and Eh measurements were taken after the supplemented TSA media were autoclaved, poured, and solidified. The following instruments were operated as per the vendor directions: (1) the a_*w*_ measurements were collected using a water activity analyzer (model 61,673,213, Rotronic Hygropalm, Rotronic Instrument Corp., Hauppauge, NY, USA); (2) pH levels were measured with the model 81074UWMMD using the probe Orion Ross Ultra pH/ATC triode meter (Thermo Fisher Scientific, Chelmsford, MA, USA); (3) EC values were measured with a model 01,301,040 Orion probe, (Thermo Scientific, Thermo Fisher Scientific, Chelmsford, MA, USA); and (4) Eh values were estimated with a model 9678BNWP Orion Sure-Flow comb Redox/ORP meter (Thermo Fisher Scientific, Chelmsford, MA, USA). The autoclaved solid media were blended using an immersion blender to a semi-liquid consistency before taking EC and Eh measurements.

## Results

The primary goals of the experiments described herein were to determine (1) if bacteria recovered from actual Mars spacecraft are capable of growth under simulated low-PTA conditions and, (2) does supplementing microbial media with anaerobic electron acceptors stimulate growth under low-PTA conditions?

To investigate the effects on growth under various incubation conditions, the following anaerobic electron acceptors for chemoorganotrophic bacteria were tested: (1) trypticase soy agar (TSA), (2) TSA + vitamins and minerals, (3) TSA + nitrate, (4) TSA + sulfate, and (5) TSA + Fe^3+^ at pH 7 or pH 5. Although the list does not include all possible anaerobic electron acceptors on Mars, it covers plausible metabolisms that might occur on Mars in the presence of organics (e.g.,^1,11,23,24^).

**Figure 3:**
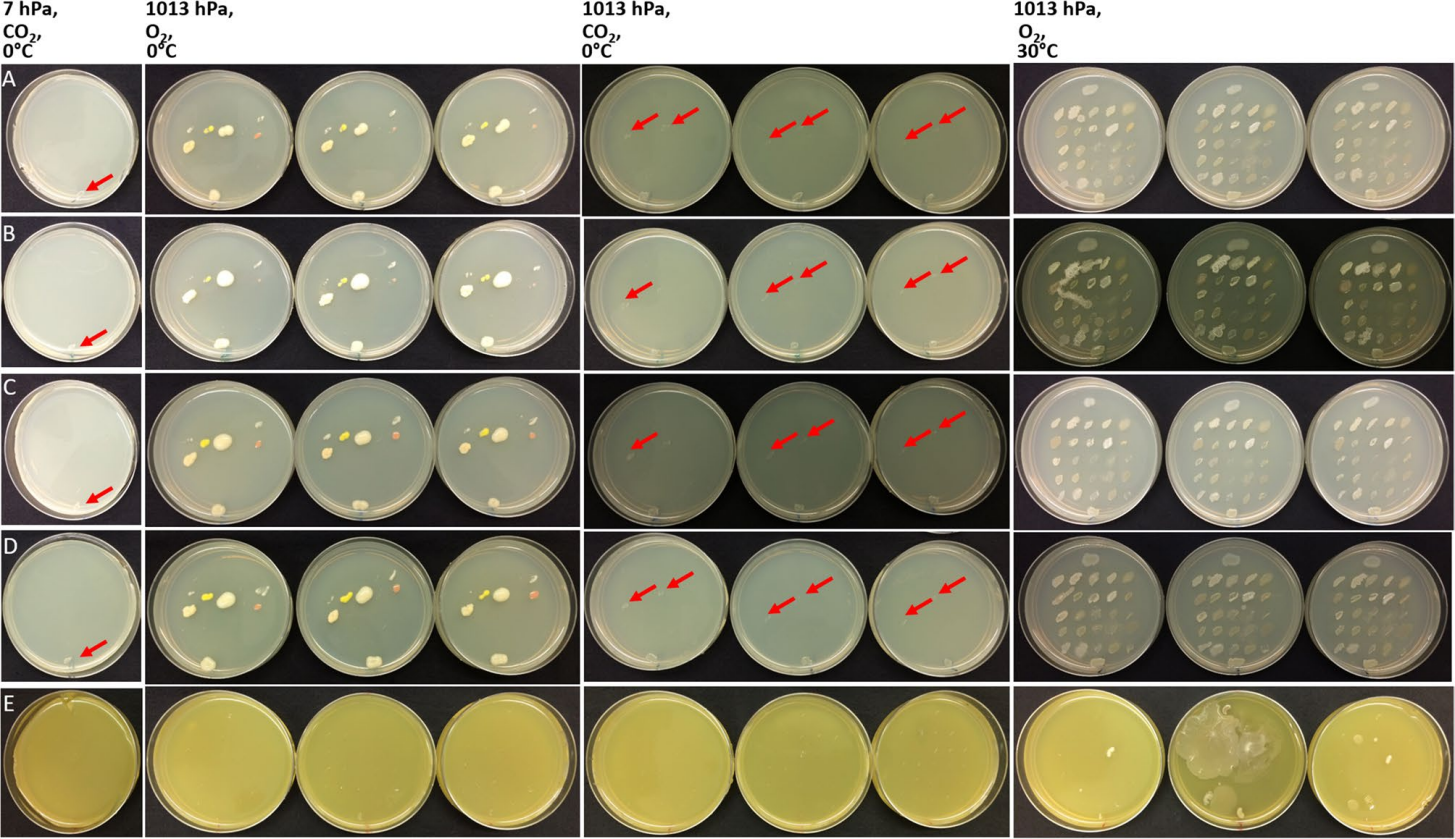
Growth of bacteria (species are listed in Fig. 2 and Table S1) on TSA plates under four different incubation conditions (top headings) and five different media (rows). A total of 25 spacecraft isolates were tested on each agar plate. All TSA plates contained the same bacteria. (**A**) The first column shows results of growth at low-PTA conditions (only one set of replicate plates are shown here); and only the positive-control, *Serratia liquefaciens* ATCC 27,592 grew (red arrows in column 1). The second set of columns shows results at 1013 hPa, 0 °C, and Earth-normal pO_2_. The third set of columns shows results for growth at 1013 hPa, 0 °C, and CO_2_-enriched anoxic atmospheres. And the fourth set of columns shows results after incubation at 1013 hPa, 30 °C, and Earth-normal pO_2_. The first three sets were incubated for 28 days, the last set was incubated for 24 h. The rows depict (**A**) TSA, (**B**) TSA + vitamins and minerals, (**C**) TSA + nitrate, (**D**) TSA + sulfate, and (**E**) TSA + Fe^3+^, pH 5. Arrows indicated positive growth with minimal microbial activity in the assays.

Figure 3 depicts the growth the strains described in Fig. 2 after 4 weeks of incubation on double thick agar plates grown under the different incubation conditions and media types. All bacteria were able to grow on TSA incubated under lab-normal conditions of 1013 hPa, 30 °C and pO_2_ (21%) and on the media supplemented with the different anaerobic electron acceptors, with the exception of the iron supplemented medium at pH 5.0 (see Fig. 3 as an example). Overall, the addition of the anaerobic electron acceptors resulted in only minor differences in colony size compared to TSA-only media with the exception of the addition of iron. Almost half of the strains (57 out of 125 strains) were not able to grow on the Fe^3+^ medium at pH 5 at optimal growth conditions (1013 hPa, 30 °C, and lab-normal pO_2_). This effect was caused by the low pH of the medium, rather than the Fe^3+^ supplement, as these strains were able to grow on the same medium at pH 7. Furthermore, it was observed that due to the lower pH, the agar surfaces were soft which led to two issues (Fig. 3E). First, it was difficult to streak the bacteria on the softened agar (i.e., the surfaces of the TSA + Fe^3+^ at pH 5.0 plates were easily damaged). And second, soft agar plates led to enhanced growth of a few bacteria that have the ability to swarm (e.g. *Sphingomonas paucimobilis*, *Micrococcus yunnanensis*, *Psychrobacillus psychrodurans*, and several bacilli). To counteract these issues, the agar concentration in the TSA + Fe^3+^ medium (pH 5.0) was increased to 3% and the number of strains per plate was reduced to four.

When comparing the different incubation conditions, clear effects of pressure, temperature, and atmospheric composition on growth were observed among the spacecraft bacteria. All strains grew under the lab-normal control conditions of 1013 hPa, 30 °C, and a lab-normal pO_2_ (21%), except as noted above for the pH 5.0 agar plates. In sharp contrast to the Earth-lab controls, only 14 of 125 bacterial strains were able to grow when the temperature was lowered to 0 °C with a lab-normal pO_2_ (Table S1). This number was reduced further to only 4 of 125 strains when the assays were incubated at 0 °C under a CO_2_-enriched anoxic atmosphere. Finally, none of the strains were able to grow under simulated low-PTA conditions (Table S1). However, all strains incubated at low-PTA conditions resumed growth when transferred to lab-normal control conditions at 1013 hPa, 30 °C, and an Earth-normal 21% pO_2_ atmosphere.

For example, the following bacteria were able to grow at 1013 hPa, 0 °C, and lab-normal pO_2_: *Staphylococcus equorum* sub. *equorum* (strains ASB-279, -309, -312), *Paenibacillus amylolyticus* (ASB-298), *Kocuria rosea* (ASB-342, -358), *Bacillus simplex* (ASB-130), *Bacillus firmus* (ASB-131), *Plantibacter flavus* (ASB-353), *Rhodococcus globerulus* (ASB-359), *Rhodococcus sp*. (ASB-356), *Psychrobacillus psychrodurans* (ASB-333), *Dietzia maris* (ASB-383), *Labdella kawkjii* (ASB-384), and *Sphingomonas yunnanensis* (ASB-391). From these 15 spacecraft bacteria, *S. equorum* sub. *equorum* strains (ASB-309 and ASB-312), and the two *Rhodococcus* strains (ASB-356 and ASB-359) were able to grow under anaerobic conditions as well. The results suggested that both low temperature and low pressure led to a reduction in the number of bacteria capable of growth. Specifically, an additive effect was observed at 1013 hPa, 0 °C and Earth-lab normal pO_2_ conditions compared to 1013 hPa and 0 °C under a CO_2_-enriched anoxic atmosphere. Adding the third stressor (i.e., low atmospheric pressure of 7 hPa) decreased the numbers of strains capable of growth to zero. No new hypopiezotolerant bacteria were recovered from the assayed 125 Mars spacecraft strains. Summarizing, the environmental conditions of low-pressure and low-temperature had a greater effect on suppressing bacterial growth than the addition of anaerobic electron acceptors did on stimulating growth.

A few changes were observed among the various strains and the five different media. One example was that two *Rhodococcus* isolates (strain ASB-356 and ASB-359) were not able to grow on TSA or TSA + Fe^3+^ pH 5 at 0 °C and CO_2_-enriched atmosphere, but colonies were observed on TSA + vitamins, TSA + nitrate, TSA + sulfate and TSA + Fe^3+^ pH 7 under the same enrichment conditions (Table S1). Similarly, *S. equorum* subsp. *equorum* (strain ASB-312) was able to grow on TSA + sulfate and possibly on TSA + nitrate at 0 °C and a CO_2_-enriched atmosphere. *Kocuria rosea* (strain ASB-358) grew better at 1013 hPa, 0 °C, and Earth pO_2_ on standard TSA when NO_3_^−^ or SO_4_^−^ were added, compared to standard TSA or TSA + vitamins. However, none of these strains were able to grow under low-PTA conditions based on the visual observations of colony sizes at the end of the 28-day assays.

In order to better quantify the effects of the added anaerobic electron acceptors under low-PTA conditions, growth rates for two hypopiezotolerant control bacteria, *S. liquefaciens* and *T. pasteurii*, were determined on four media (e.g., TSA + Fe^3+^ pH 5 medium was excluded). In addition, four representative spacecraft bacteria, that exhibited negative growth on the visually evaluated agar assays, were chosen to determine if their growth rates were so low that they were rated as negatives on the TSA assays while in fact they were true hypopiezotolerant bacteria, albeit with extremely slow growth rates. *Staphylococcus equorum* subsp. *equorum* (ASB-279) was incubated on TSA + vitamins, *B. simplex* (ASB-130) was incubated on TSA + Fe^3+^citrate and sodium lactate at pH 7, *A. johnsonii* (ASB-326) was incubated on TSA + vitamins, and *S. yunnanensis* (ASB-391) was incubated on TSA + sulfate. Aliquots (2.5 µl) of the cell suspensions were applied on each of the media tested, and incubated at low-PTA conditions for 28 days. Every 3–4 days, three random samples were collected by aseptically cutting out a small area of TSA agar containing the cells.

The shortest doubling time for the hypopiezotolerant control strain, *S. liquefaciens*, was observed when the medium was supplemented with sulfate, and the slowest growth rate was observed for the iron-supplemented medium (Table 1, Fig. 5). A lag phase of up to 7 days was observed for all other media tested. After 28 days of incubation at low-PTA conditions, the lowest cell densities were reached when *S. liquefaciens* cells were grown on TSA + nitrate and TSA + Fe^3+^, pH 7. The final cell densities of *S. liquefaciens* on TSA, TSA + sulfate, and TSA + vitamins were approx. 1 to 1.5 logs higher compared to the nitrate and iron supplemented media.

**Table 1:** Growth rates (GR; per day) and doubling times (DT; days) for the two hypopiezotolerant strains *Serratia liquefaciens* and *Trichococcus pasteurii*, and four spacecraft bacteria *Staphylococcus equorum* subsp. *equorum*, *Acinetobacter johnsonii*, *Sphingomonas yunnanensis*, and *Bacillus simplex*. The data were calculated from the plots in Fig. 4, 5, and 6, respectively.

Isolates	TSA		TSA + vitamins		TSA + nitrate		TSA + sulfate		TSA + iron pH 7	
	GR per day	DT days	GR per day	DT days	GR per day	DT days	GR per day	DT days	GR per day	DT days
*S. liquefaciens*	0.21	3.39	0.19	3.57	0.23	2.99	0.15	4.70	0.11	6.14
*T. pasteurii*	0.22	3.18	0.25	2.83	0.25	2.77	0.36	1.91	0.17	4.04
*S. equorum*	–	–	− 0.06	− 12.56	–	–	–	–	–	–
*A. johnsonii*	–	–	− 0.11	− 6.45	–	–	–	–	–	–
*S. yunnanensis*	–	–	–	–	− 0.02	− 29.00	–	–	–	–
*B. simplex*	–	–	–	–	–	–	–	–	− 0.25	− 2.415

**Figure 4:**
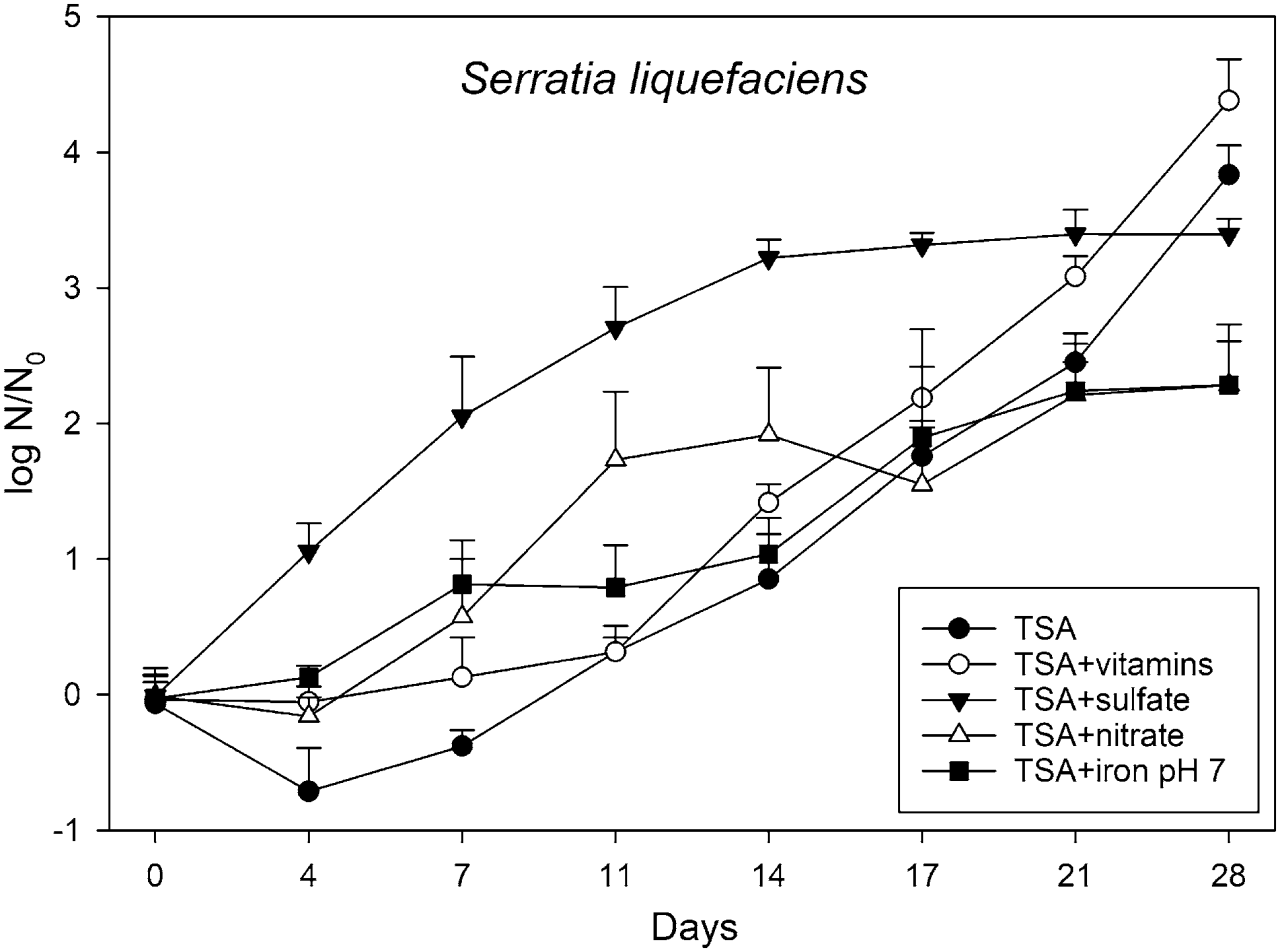
Effects of the different anaerobic electron acceptors on the growth of *Serratia liquefaciens* on TSA, TSA + vitamins, TSA + nitrate, TSA + sulfate, and TSA + Fe^3+^, pH 7 at low-PTA conditions. Samples were taken at different incubation times up to 28 days. Cells were applied to the agar plates as multiple, 2.5-µL drops (~ 2.5 × 10^5^ cells/drop), squares of agar/cells were cut out, and subsequent MPN assays completed to estimate the numbers of viable cells over time. The data are reported as log10 (N/N_0_) in which N_0_ = viable cell numbers at T = 0 and N = viable cell numbers at a specific time-step between 3 and 28 days (n = 6; error bars are standard deviations).

**Figure 5:**
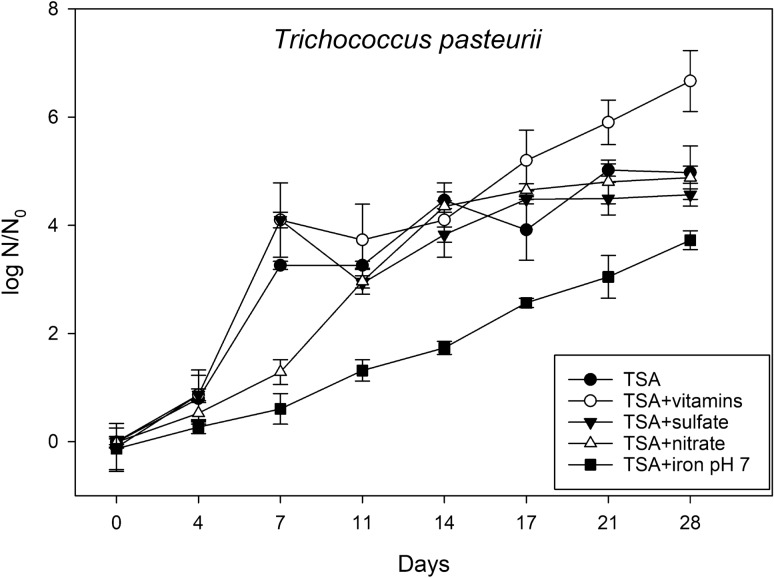
Effects of the different anaerobic electron acceptors on the growth of *Trichococcus pasteurii* on TSA, TSA + vitamins, TSA + nitrate, TSA + sulfate, and TSA + Fe^3+^ at pH 7 under low-PTA conditions. Samples were taken at different incubation times up to 28 days. Cells were applied to the agar plates as multiple, 2.5-µL drops (~ 2.5 × 10^5^ cells/drop), squares of agar/cells were cut out, and subsequent MPN assays completed to estimate the numbers of viable cells over time. The data are reported as log10 (N/N_0_) in which N_0_ = viable cell numbers at T = 0 and N = viable cell numbers at a specific point between 3 and 28 days (n = 6; error bars are standard deviations).

**Figure 6:**
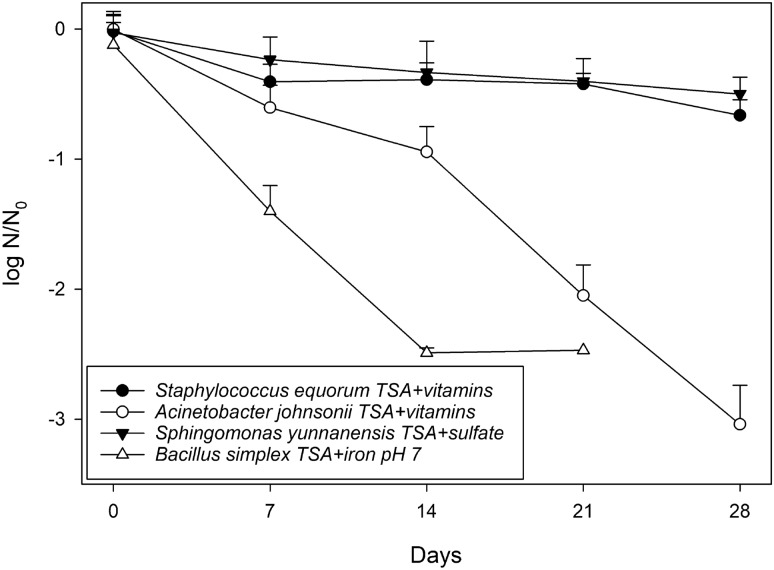
Exposure of four spacecraft strains to low-PTA conditions on different medium types. The four species were *Staphylococcus equorum* subsp. *equorum*, *Acinetobacter johnsonii*, *Sphingomonas yunnanensis*, and *Bacillus simplex*. The media tested were TSA + vitamins, TSA + sulfate, and TSA + Fe^3+^ at pH 7, in which plates were incubated at low-PTA conditions for up to 28 days. Samples were taken every 7 days. Cells were applied on agar plates as 2.5-µL drops (~ 2.5 × 10^5^ cells/drop), squares of agar/cells were cut out, and subsequent MPN assays completed. The data are reported as log 10 (N/N_0_) in which N_0_ = viable cell number on at T = 0 time, and N = viable cell numbers at specific points after incubation from 7 to 28 days (n = 6; error bars are standard deviations).

A similar trend was observed for *T. pasteurii* with the exception that the fastest doubling rate was determined for TSA + nitrate (Table 1). The highest cell density was reached on theTSA + vitamins medium, the lowest cell number was revealed on TSA + Fe^3+^ supplemented medium at pH 7.0 (Fig. 5). As seen for *S. liquefaciens,* a plateau was reached for the TSA + sulfate medium after 17 days. However, compared to *S. liquefaciens* higher overall cell densities were observed for all *T. pasteurii* cultures incubated for 28 days in all media tested.

In contrast, the four tested strains originating from Mars spacecraft revealed a decrease in viable cell numbers compared to the initial inoculation density (Fig. 6). In fact, a 2.5–3 log reduction was observed for *B. simplex* and *A. johnsonii* at 28 days*,* while *S. equorum* subsp. *equorum,* and *S. yunnanensis* were reduced only by half a log after 28 days.

Water activity (a_*w*_), pH, EC, and Eh measurements (Table 2) were recorded for the media tested because these data might provide an indication whether there are geochemical constraints that have an influence on microbial growth of spacecraft bacteria under low-PTA conditions. Some minor fluctuations for EC and Eh were observed among the media, while a_*w*_ and pH levels were similar across all of the media tested in the assays. Thus, there were no clear effects of a_*w*_ and pH parameters on growth in the agar-based assays (Fig. 3, Table S1) and the growth assays (Figs. 4, 5, 6). In contrast, growth-limiting conditions were likely caused by atmospheric pressure (1013 vs 7 hPa), temperature (0 vs 30 °C), and pO_2_ (lab-normal versus low pO_2_ in the low-PTA conditions) in the low-pressure desiccators (Fig. 3 and Table S1).

**Table 2:** Water activity (a_*w*_), pH, Eh, and EC measurements for the anaerobic electron acceptors amended media after the media was autoclaved and solidified.

Measurement	Units	TSA	TSA + vitamins	TSA + nitrate	TSA + sulfate	TSA + Fe (pH 7)
a_*w*_		0.980	0.982	0.981	0.980	0.982
EC	mS/cm	4.97	4.70	6.41	8.92	–
Eh	mV	170.9	164.2	150.2	156.8	136.9
pH		7.08	7.02	7.07	6.97	7.27

## Discussion

The survival, metabolism, and growth of terrestrial microorganisms under Martian conditions should be characterized in order to predict the potential propagation of Terran life on Mars during future surface operations. To prevent forward contamination, mission objectives must be consistent with planetary protection guidelines for landers and rovers that are subject to strict cleaning and monitoring protocols^7,8,18^. Currently, the required threshold for non-life detection Category IVa landers is < 300 bacterial spores per square meter and < 3 × 10^5^ spores per spacecraft prior to launch^7^. Because the bioburden thresholds are not zero, some viable microorganisms are launched on Mars-bound surface spacecraft where they face a variety of biocidal or inhibitory factors. Amongst these are low pressure, low temperature, and an anoxic atmosphere.

Recently, approx. 30 species of bacteria from 10 genera were described that are capable of growth at low pressures ≤ 10 hPa^12,14–17^ and defined as hypopiezotolerant bacteria by Schwendner and Schuerger^16^. These experiments were conducted under simulated low-pressure Martian conditions of 7 hPa, 0 °C, and a CO_2_-enriched anoxic atmosphere (i.e., low-PTA conditions). However, the current identified hypopiezotolerant bacteria, have been isolated primarily from extremophilic ecosystems including permafrost, arctic, and alpine niches. One notable exception is the demonstration that the mesophilic bacterium, *S. liquefaciens*, can grow under low-PTA conditions^12,15^.

To identify whether mesophilic culturable bacteria prevalent on Mars spacecraft will pose a risk to forward contamination of the surface, the growth of 125 bacteria recovered directly from six authentic Mars spacecraft was tested under low-PTA conditions with a focus on investigating the effect of simulated Martian atmospheric pressure and media augmentation with anaerobic electron acceptors. In previous studies with arctic, alpine, permafrost samples^14,15^, the majority of bacteria were found incapable of growth under low-PTA conditions, even though adequate water and nutrients were provided using the organic rich medium TSA. In the current study, we sought to expand the list of media and include a diverse set of anaerobic electron acceptors that might power anaerobic heterotrophic metabolisms such as nitrate, sulfate, or ferric iron reduction; plus the nutrients available in TSA.

Two key findings of the current study suggest that naturally occurring culturable mesophilic, organotrophic spacecraft bacteria may not be able to thrive on Mars once transferred to the surface. First, none of the 125 bacteria tested were capable of growth under low-PTA conditions suggesting a low risk of clean room chemoorganotrophic bacteria being able to proliferate on the surface of Mars. Furthermore, even when hypopiezotolerant bacteria are able to grow under low-PTA conditions (e.g., *S. liquefaciens*, *T. pasteurii*^12,15–17^) the assays had to be continuously maintained under stable hydrated conditions with rich sources of organics, for at least 10–14 days, before growth was observable. Such stable liquid and nutrient-rich conditions are very unlikely to occur on the surface of current-day Mars^1,10^, and thus, actual conditions on Mars are likely to be significantly more biocidal or inhibitory for the bacteria tested here. Although ‘…*follow the water*…’ has become a paradigm for the search for life on Mars, we should not exclude the significant role that environmental conditions in the bulk atmosphere on Mars play on defining habitable regions near the surface.

And second, the added anaerobic electron acceptors failed to stimulate the chemoorganotrophic bacteria tested here that represent a portion of the naturally occurring bioburden on authentic Mars spacecraft. Thus, it seems unlikely that additional geochemical redox couples on Mars will be able to overcome the inherent metabolic, genomic, transcriptomic, and proteomic constraints on many spacecraft microorganisms. This finding was unexpected because numerous papers on the habitability of Mars have suggested that geochemical redox couples are likely present on Mars, with the potential to support not only lithotrophic but also organotrophic growth (see^25^ and reviews by^1,11,13^). However, the current study cannot rule out all possible chemoorganotrophic metabolisms on Mars because we only focused here on naturally occurring culturable spacecraft bacteria recovered during routine planetary protection monitoring procedures. Currently these are the only isolates available that were directly recovered from Mars-bound spacecraft. There have been few cultivation attempts that targeted psychrophiles, anaerobes, or other microbial specialists from spacecraft.

There are two potential reasons for the ineffectiveness of the added anaerobic electron acceptors to stimulate bacterial growth under low-PTA condition. First, almost all described hypopiezotolerant bacteria to date were isolated from arctic, permafrost, and alpine soils, with the exception of *S. liquefaciens*^12,15^. The hypopiezotolerant bacteria from these cold environments are adapted to low temperatures, contrary to the clean room environments used for spacecraft assembly in which the temperatures do not drop below 20 °C. The fact, that the mesophilic, heterotrophic bacterium, *S. liquefaciens,* was able to grow under low-PTA conditions was initially unexpected^12^, and therefore highlights the importance of testing other mesophilic species. This is particularly relevant with regard to planetary protection because culturable mesophilic bacteria are likely to remain a key group of microorganisms targeted for pre-launch microbial assays^7,8,18^. Based on the results of our study, it appears that low temperature and low pressure inhibited growth more than the augmented anaerobic electron acceptors could stimulate growth.

Second, clean room assays for satisfying planetary protection guidelines are generally conducted under aerobic conditions^7,8^, and therefore target only a small diversity of the overall culturable microbiota on spacecraft. This longtime proven approach focuses on enumerating spore-forming bacterial species as proxies for estimating the total bioburdens on spacecraft. Only a few studies have described the abundance of anaerobic versus obligate-aerobic bioburdens on Mars spacecraft. For example, studies from a spacecraft clean room in Kourou, French Guiana have shown that anaerobic sampling and subsequent anoxic enrichments can lead to a higher microbial diversity, including the recovery of facultative anaerobes and extremophilic bacteria compared to only applying the standard aerobic assay protocol^26–28^. Thus, the species diversity used in the current study may have been partially biased to aerobic, mesophilic, fast-growing species as a result of the isolates being obtained from the standardized planetary protection protocols and existing archives. However, 19 of the 36 genera tested included isolates that have been described as facultative anaerobes. These include species of *Bacillus*, *Corynebacterium*, *Exiguobacterium*, *Janibacter*, *Lysinibacillus*, *Methylobacterium*, *Microbacterium*, *Micrococcus*, *Oceanobacillus*, *Paenibacillus*, *Pantoea*, *Paracoccus*, *Pseudomonas*, *Ralstonia*, *Rhodococcus*, *Serratia*, *Sporosarcina*, *Staphylococcus*, and *Streptococcus*^29^.

Furthermore, only recently the microbiome of spacecraft and their surrounding clean rooms have been investigated using cutting edge omics technologies such as next generation sequencing or shotgun metagenome sequencing^30–33^. These recent studies have used propidium monoazide (PMA) to bind to DNA from dead bacteria or from free extracellular DNA. Thus, PMA-treated samples allow the differentiation between live and dead cells, and therefore provide information for characterizing the active microbiome on spacecraft. The application of PMA revealed that more than 90% of the total microbial signatures found on spacecraft or SAF floors originated from dead bacteria or free extracellular DNA^34^.

In line with the cultivation studies revealing facultative anaerobes, metagenomic studies of PMA-treated samples have found genetic evidence for fermentive and respiratory metabolic pathways in the living spacecraft microbiome. The fact that Weinmaier et al.^31^ reported the absence of carbon, nitrogen, and sulfur cycling from samples collected from clean room floors in PMA-treated samples is surprising since nitrate reduction has been reported for common spacecraft contaminants like *Bacillus*^35^ and *Paenibacillus*^36^. To obtain an in-depth knowledge of the overall metabolic capability of an active clean room microbiome, more metagenomics data from actual Mars spacecraft are required.

When selecting the bacterial strains for the current study, we chose strains from several dominant families represented in the literature as being recoverable from Mars spacecraft (e.g.,^5,27,28,37–39^), and two species belonging to genera with known hypopiezotolerant strains^15^. Examples of genera that include hypopiezotolerant species are: *Bacillus*, *Paenibacillus*, *Rhodococcus*, *Streptomyces*, *Exiguobacterium,* and *Serratia*. It is plausible that the aerobic spacecraft screening procedure and the selection of the isolates to be tested led to an underestimation of species capable of growth under simulated low-PTA conditions despite the fact that facultative anaerobic isolates were recovered during the spacecraft assays.

For example, *S. equorum* subsp. *equorum*, and two *Rhodococcus* spp., were able to grow under an anoxic atmosphere at 0 °C (Table S1). It is generally known that *Staphylococcus* spp. are facultative anaerobes^40^. In this study we tested 13 *Staphylococcus* strains in eight species, but only two isolates grew under anoxic conditions suggesting that the ability to grow under anoxic conditions was adversely affected by low temperature. Adding low pressure conditions, which are not naturally occurring on Earth, to low temperature and high pCO_2_, none of the *Staphylococcus* strains were able to grow indicating an interactive, complex response of the three stressors which led to growth inhibition of these two species, and for all 125 spacecraft bacteria tested.

In contrast, although the 125 spacecraft bacteria were not able to thrive under low-PTA conditions, but resumed growth at optimal enrichment conditions, cells might remain in a viable state with the potential of active metabolism after the cells are returned to conducive conditions. Upon return of the tested strains to lab-normal conditions of 1013 hPa, 30 °C, and 21% pO_2_, growth of all strains resumed indicating that low-PTA conditions were reversible and not lethal. Similar results were reported for spacecraft^12^ and non-spacecraft^14,15,17^ bacterial species that were initially inhibited under low-PTA conditions.

Within the current experimental protocols, it is unknown if the strains were metabolically active but not growing during the low-PTA assays or whether their growth rates were too slow to be detectable after 28 days. In general, doubling times for bacteria increase as the temperature is lowered, especially for mesophilic bacteria that are not well adapted to low temperatures. The incubation time used here cannot be prolonged with the current agar-based assays as the agar plates start to desiccate after 28 days. Therefore, other measurements of metabolic activity and growth are required to rule out very slow growth rates under low-PTA conditions.

A more precise quantitative assay was applied to determine if the cells from four spacecraft bacteria were growing too slow to be undetectable by the visual observation of colonies on the TSA-based agar assays at low-PTA conditions. All four strains (*S. equorum* subsp. *equorum*, *A. johnsonii*, *S. yunnanensis*, and *B. simplex*) failed to increase their cell densities over 28 days (Fig. 6) confirming that the cells were unable to grow under low-PTA conditions. In fact, all four species exhibited cell death as confirmed by 0.5- to 3-log reductions in numbers over the course of 28 days. Thus, increased confidence was acquired that our initial results with visual observations on TSA-based assays did indeed capture the non-growth of 125 bacteria strains incubated under low-PTA conditions.

## Conclusion

Using the low-PTA assays, we have screened 125 bacteria recovered from six authentic Mars spacecraft. None of the bacteria tested were confirmed as hypopiezotolerant microbes capable of growth under simulated low-pressure Martian conditions. These results indicate that a range of the chemoorganotrophic and mesophilic bacteria on Mars spacecraft cannot easily grow under simulated conditions of low pressure, low temperature, and CO_2_-enriched anoxic atmospheres, even if stable hydrated and nutrient-rich conditions are provided. Furthermore, the addition of various anaerobic electron acceptors to the TSA-based media did not significantly promote growth under low-PTA conditions. Results suggest that many of the culturable chemoorganotrophic bacteria present on spacecraft at launch may not pose a significant risk for potential proliferation once transferred to Mars. Although we tested the effects of only three environmental factors and five different TSA media on the growth of 125 spacecraft bacteria under simulated low-PTA conditions, the case for the proliferation of spacecraft bacteria on Mars is less likely than described here because other biocidal and inhibitory factors are present on Martian surface (see^1,10^). For example, up to 22 biocidal and inhibitory factors have been discussed in papers on the habitability of the Martian surface (e.g.,^1,10–13^). Many of these biocidal factors (e.g., solar UVC irradiation, extreme desiccation in the regolith, volatile oxidizing agents, high salt concentrations) would likely further inhibit cell growth if added as cofactors to the experiments reported here. We conclude that the probability of growth may be low on Mars for a wide diversity of culturable chemoorganotrophic bacteria prevalent on spacecraft prior to launch, and that the habitability of the modern Martian surface is likely to be significantly constrained by the harsh biocidal and inhibitory environmental factors present on the surface.

## Supplementary information

Supplementary Table S1.

Supplementary Table S2.

Supplementary Legends.

## Data Availability

The datasets for the growth curves in Figs. 4, 5, and 6 are provided as an Excel file in the supplemental data for this paper (Table S2). Furthermore, all raw data will be posted in the University of Florida Institutional Repository (UFIR) at the website https://ufdc.ufl.edu.ufirg within 3 months of the publication of the study. Search for Andrew Schuerger and then the title of the paper.
