# Supplementary material for: Addition of anaerobic electron acceptors to solid media did not enhance growth of 125 spacecraft bacteria under simulated low-pressure Martian conditions
Source: Sci Rep. 2020 Oct 26;10:18290. doi: 10.1038/s41598-020-75222-2 (PMC7588431; doi:10.1038/s41598-020-75222-2)
Supplement: Supplementary file 3 — Supplementary Legends. [file 41598_2020_75222_MOESM3_ESM.docx]

Supplementary Information for:

**Addition of Anaerobic Electron Acceptors to Solid Media did not Enhance Growth of 125 Spacecraft Bacteria under Simulated Low-Pressure Martian Conditions**

**Petra Schwendner^1*^, Mary-Elisabeth Jobson^1^, Andrew C. Schuerger^1^**

^1^ University of Florida, 505 Odyssey Way, Space Life Sciences Lab, Exploration Park, Merritt Island, FL 32953

*** Correspondence:**Petra Schwendner
Petra.Schwendner@ufl.edu

**Supplementary Table S1:** Growth results of the 125 bacteria isolates tested under (A) 1013 hPa, 30°C, O2; (B) 1013 hPa, 0°C, O2; (C) 1013 hPa, 0°C, CO2; and (D) low-PTA conditions: 7 hPa, 0°C, CO2. Isolates that have been rated positive when colonies have been observed in ≥ 2 plates are displayed. [(+) = positive growth, (‒) = negative growth]; n = 3. The first column lists the lab identifier code. The second column provides the strain identity based on 16S rRNA gene sequencing, the third column lists the sample the isolate originates from. The following columns list the different incubation conditions and the different media tested. NA = not available

**Supplementary Table S2**: Raw data and the equations of the linear fitted models for the bacterial growth curves in Figures 4, 5, and 6. Each dataset is given as a separate sheet in the Excel file.
